# An interpretable machine learning model for predicting brain metastasis in breast cancer

**DOI:** 10.3389/fmed.2026.1693557

**Published:** 2026-04-08

**Authors:** Hong Wang, Hui Zhang, Meng Chang, Jingjing Chen, Binxu Qiu, Chao Yang, Chao Gao

**Affiliations:** 1 Department of Breast Surgery, First Ward, Tangshan People’s Hospital, Tangshan, Hebei, China; 2 Hebei Key Laboratory of Molecular Oncology, Tangshan, Hebei, China; 3 Breast Center, West China Hospital, Sichuan University, Chengdu, China; 4 Department of Breast Center, The Fourth Hospital of Hebei Medical University, Shijiazhuang, China; 5 Department of Radiotherapy, The Fourth Hospital of Hebei Medical University, Shijiazhuang, China

**Keywords:** brain, breast cancer, machine learning, metastasis, model

## Abstract

**Background:**

Breast cancer is the most common malignancy worldwide. Brain metastasis in breast cancer severely impacts prognosis, and the objective of this study is to develop a machine learning model for predicting the risk of brain metastasis in breast cancer patients to assist clinical management.

**Methods:**

Univariate and multivariate logistic regression analyses were employed to screen the final included variables, and eight machine learning algorithms were utilized for model construction. Model performance was evaluated using receiver operating characteristic curves, precision-recall curves, decision curve analysis (DCA), and calibration curves, with the optimal model selected based on these metrics. The model was trained on a cohort of 154,193 patients, internally validated on 66,084 patients, and externally validated on 765 real-world cases, incorporating metrics such as area under the curve (AUC), area under the precision-recall curve (AUPRC), decision curves, and calibration plots, while SHAP analysis was applied to enhance interpretability. A web-based calculator was developed based on the optimal model to facilitate clinical application.

**Results:**

Univariate logistic regression identified higher tumor grade, advanced T/N stage, advanced clinical stage, and PR positivity as risk factors, whereas radiotherapy, chemotherapy, surgery, HR + /HER2- subtype, and unilateral tumors served as protective factors (*P* < 0.001). Multivariate analysis confirmed independent risk factors, including poorer pathological grade, N3 lymph node status, later stage, and PR positivity, and protective factors, including radiotherapy, chemotherapy, surgery, non-HR-/HER2- subtypes, and HER2 positivity. The XGBoost model achieved an AUC of 0.98 in 10-fold cross-validation, with AUCs of 0.99 and 0.97 in the internal test set and external validation set, respectively; AUPRC values were 0.933, 0.864, and 0.648; decision curve analysis demonstrated superior net benefit compared to alternative models within the 0.1–0.8 threshold range; calibration curves showed high concordance between predicted and observed event rates. SHAP analysis highlighted surgery as the primary protective factor, followed by stage and T classification as risk enhancers, revealing interactions among treatment variables.

**Conclusion:**

This study developed an interpretable and clinically deployable XGB model, accompanied by a web-based calculator, thereby advancing personalized risk stratification, early screening, and resource optimization in the management of breast cancer brain metastasis.

## Introduction

Breast cancer (BC) remains one of the most prevalent malignancies globally, posing a substantial public health challenge. Its incidence is alarmingly increasing by 1–5% annually in many countries, particularly among younger women under 50 (1). Among the most devastating complications of breast cancer is brain metastasis (BM). BM occurs in a substantial proportion of patients with metastatic breast cancer and is more common in aggressive subtypes. In HER2-positive metastatic breast cancer, studies have reported that approximately 30–50% of patients develop central nervous system metastases over the course of the disease (2). Triple-negative breast cancer also shows a high propensity for BM (3). BM profoundly impacts patient prognosis and quality of life, typically manifesting 2–3 years after initial diagnosis with severe neurological deficits and high mortality rates (4). The prognosis is particularly grim due to limited treatment options and the impediment of the blood-brain barrier to systemic therapies, resulting in a median overall survival of only 2.9–9 months post-diagnosis, with merely 20% of patients surviving beyond 1 year (5, 6). Therefore, early identification and intervention for BM are paramount, necessitating the development of accurate predictive models for individualized risk assessment, timely screening, and targeted therapies, which can ultimately improve patient survival and quality of life, and aid in prognostic stratification and resource allocation (7).

While prior research has explored predictive models for BCBM, several significant limitations persist. These include reliance on single-institution cohorts, lack of external validation for models built on public databases, issues of overfitting, limited integration of diverse features, and inadequate handling of heterogeneous data, often leading to suboptimal performance in real-world settings (8, 9). Furthermore, previous studies frequently overlooked comprehensive BM risk factors or failed to adequately address biases inherent in metastatic cancer datasets, thereby hindering the models’ ability to fully capture the complexity of BM progression (10). To address these challenges, we used SEER data to construct a population-based cohort and conducted downstream statistical and machine-learning analyses to identify predictors of brain metastasis. As a comprehensive, population-based repository covering approximately 28% of the U.S. population, SEER provides detailed, high-quality data on cancer incidence, staging, treatment, and long-term survival. Its active follow-up from diagnosis to death ensures robust, representative data spanning diverse demographics, enabling generalizable findings and the exploration of long-term trends (11–13). Moreover, its capability to link with other datasets further enhances the analytical scope for health outcomes, positioning SEER as an invaluable resource for developing robust and generalizable predictive models (14).

Against this background, the present study aimed to develop and validate robust machine learning models for predicting brain metastasis risk in breast cancer patients, leveraging the extensive SEER database. We incorporated a broader range of clinical variables and employed rigorous cross-validation techniques to enhance model accuracy and applicability. Crucially, our models were further validated using real-world data to ensure superior generalization capability and clinical utility, providing a quantifiable tool for clinical decision-making. This research holds significant clinical implications, as accurate prediction of BCBM can guide personalized surveillance strategies, facilitate early therapeutic interventions, and ultimately reduce mortality rates in this high-risk patient population.

## Materials and methods

### Study population and inclusion and exclusion criteria

This retrospective study utilized data from the SEER database (2010–2022) and a Chinese medical institution. Given that no personally identifiable information was used, patient informed consent and institutional review board approval were not required for this retrospective analysis (Supplementary Tables 1, 2). Patients diagnosed with breast cancer were extracted from the SEER database and subsequently divided into training and internal validation cohorts using a 7:3 ratio. The inclusion criteria for SEER database patients were: (1) pathologically confirmed breast cancer diagnosis; (2) absence of concurrent malignancies; and (3) availability of complete clinical information, including age, sex, race, marital status, histological grade, tumor size, T stage, N stage, and breast cancer subtype. Patients were excluded if they: (1) were diagnosed before 2010; (2) had incomplete clinical information; or (3) presented with another primary malignancy. All included cases featured comprehensive clinicopathological data and follow-up information without evidence of other primary tumors. Demographic and clinicopathological variables collected from both cohorts included: race, age, sex, primary site, laterality, T stage, N stage, M stage, surgical intervention, radiotherapy, chemotherapy, bone metastasis, and survival duration. The external validation cohort consisted of breast cancer patients from Tangshan People’s Hospital, collected between 2021 and 2024. The study was approved by the Ethics Committee of the hospital and conducted in accordance with the principles of the Declaration of Helsinki. To maintain consistency with SEER database classification, patients from the Chinese medical institution were categorized as “Other” under the race variable.

### Machine learning model construction

This study employed univariate and multivariate logistic regression analyses to evaluate risk factors associated with brain metastasis in the training cohort. Odds ratios (OR) with 95% confidence intervals (CI) were calculated, with OR > 1 defined as indicating potential risk factors. Statistical significance was set at *P* < 0.05. Variables demonstrating statistical significance in multivariate logistic regression analysis were subsequently incorporated as input features for machine learning model development. The machine learning methodology was implemented using Python (version 3.10) and the scikit-learn library (version 0.24). The dataset was randomly partitioned into training and testing sets at a 7:3 ratio. Eight distinct machine learning algorithms were selected for model construction, including: Logistic Regression (LR) (15), Gradient Boosting Machine (GBM) (16), Extreme Gradient Boosting (XGB) (17), Random Forest (RF) (18), Light Gradient Boosting Machine (LGB) (19), Decision Tree (DT) (20), and Bernoulli Naive Bayes (BNB) (21). To optimize model performance, hyperparameter tuning was conducted using the random search method. Model stability was assessed through internal validation performed on the training set using 10-fold cross-validation. The predictive performance of each model was comprehensively evaluated on the testing set using multiple metrics, specifically: area under the receiver operating characteristic curve (AUC), area under the precision-recall curve (AUPRC), decision curve analysis (DCA), and calibration curves. This multi-faceted evaluation strategy ensured a comprehensive and reliable assessment of model discrimination, prediction accuracy, clinical utility, and calibration.

### Interpretable machine learning

The inherent opacity of machine learning model decision-making processes makes it challenging for users to discern how input data influences predicted outcomes. To mitigate this challenge, we introduced Shapley Additive Explanations (SHAP) to achieve instance-level interpretability (22). Grounded in cooperative game theory, SHAP quantifies each feature’s marginal contribution to an individual prediction relative to a reference baseline, thereby decomposing model outputs into additive feature effects. Utilizing the Python SHAP package, we generated global and local visualizations of feature importance, dependence, and interactions. This approach significantly enhanced model transparency, successfully transforming the optimized model from a “black box” into an interpretable clinical tool. It clearly revealed the intrinsic relationships between key risk factors and predicted outcomes, which is expected to significantly boost clinicians’ confidence and the practical feasibility of its implementation.

### Statistical analysis

Data processing and statistical analyses were performed using R software (version 4.0.5). The normality of continuous variables was assessed using the Shapiro-Wilk test. Normally distributed continuous variables were presented as mean ± standard deviation (SD) and compared between groups using the independent samples *t*-test. Non-normally distributed continuous variables were expressed as median with interquartile range and analyzed using the Mann-Whitney U test. Categorical variables were summarized as frequencies and percentages and compared using the chi-square test. All statistical tests were two-sided, with *P* < 0.05 considered statistically significant. This comprehensive analytical approach enabled appropriate handling of data distributions and ensured robust statistical comparisons between study groups.

## Results

### Baseline characteristics

The baseline characteristics of patients with breast cancer are summarized in Table 1. In the training cohort (*N* = 154,193), internal validation cohort (*N* = 66,084), and external validation cohort (*N* = 765), the majority of patients were aged older than 50 years, with females comprising more than 99% of cases in all cohorts. Compared with the training and internal validation cohorts, the external validation cohort exhibited a higher proportion of patients who did not receive radiotherapy (63.1% vs. 49%) or chemotherapy (77.1% vs. 60%). Married patients predominated in the training and internal validation cohorts (55.8 and 55.5%, respectively), whereas unmarried patients were more prevalent in the external validation cohort (52.9%). The vast majority of patients underwent surgery (92%), and histological grade II was the most common (44%). Across all cohorts, T1 and N0 were the most frequent primary tumor and lymph node stages, respectively. Stage I disease was the most prevalent overall (52%), whereas stage IV disease accounted for less than 3% in each cohort. The predominant molecular subtype was HR + /HER2- (approximately 75%), with ER and PR positivity rates of approximately 84 and 74%, respectively, and a HER2 positivity rate of around 14%. Tumor laterality was evenly distributed between the right and left breasts in the training and internal validation cohorts, whereas the external validation cohort demonstrated a substantially higher proportion of bilateral cases (53.3%) and no left-sided cases. The overall incidence of brain metastases was low.

**TABLE 1:** Baseline characteristics of breast cancer patients in training, internal validation, and external validation cohorts.

Variables	Training	Internal validation	External validation
	*N* = 66,084	*N* = 154,193	*N* = 765
Age			
≤50	13213 (20.0%)	30860 (20.0%)	34 (4.44%)
50	52871 (80.0%)	123333 (80.0%)	731 (95.6%)
Sex			
Male	467 (0.71%)	1058 (0.69%)	4 (0.52%)
Female	65617 (99.3%)	153135 (99.3%)	761 (99.5%)
Radiation			
No	32668 (49.4%)	76305 (49.5%)	483 (63.1%)
Yes	33416 (50.6%)	77888 (50.5%)	282 (36.9%)
Chemotherapy			
No	39672 (60.0%)	93321 (60.5%)	590 (77.1%)
Yes	26412 (40.0%)	60872 (39.5%)	175 (22.9%)
Marriage			
No	29220 (44.2%)	68650 (44.5%)	405 (52.9%)
Yes	36864 (55.8%)	85543 (55.5%)	360 (47.1%)
Surgery			
No	3636 (5.50%)	8349 (5.41%)	54 (7.06%)
Yes	62448 (94.5%)	145844 (94.6%)	711 (92.9%)
Grade			
I	16762 (25.4%)	39120 (25.4%)	227 (29.7%)
II	29250 (44.3%)	68266 (44.3%)	364 (47.6%)
III	20018 (30.3%)	46701 (30.3%)	174 (22.7%)
IV	54 (0.08%)	106 (0.07%)	0 (0)
T			
1	39717 (60.1%)	93189 (60.4%)	491 (64.2%)
2	20001 (30.3%)	46451 (30.1%)	212 (27.7%)
3	4085 (6.18%)	9276 (6.02%)	33 (4.31%)
4	2281 (3.45%)	5277 (3.42%)	29 (3.79%)
N			
0	45798 (69.3%)	107225 (69.5%)	595 (77.8%)
1	14902 (22.6%)	34545 (22.4%)	129 (16.9%)
2	3298 (4.99%)	7590 (4.92%)	22 (2.88%)
3	2086 (3.16%)	4833 (3.13%)	19 (2.48%)
Stage			
I	34479 (52.2%)	80864 (52.4%)	446 (58.3%)
II	22161 (33.5%)	51723 (33.5%)	252 (32.9%)
III	7155 (10.8%)	16269 (10.6%)	51 (6.67%)
IV	2289 (3.46%)	5337 (3.46%)	16 (2.09%)
Breast-subtype			
HR-/HER2-	7045 (10.7%)	16479 (10.7%)	76 (9.93%)
HR-/HER2 +	2816 (4.26%)	6512 (4.22%)	24 (3.14%)
HR + /HER2-	49548 (75.0%)	115586 (75.0%)	611 (79.9%)
HR + /HER2 +	6675 (10.1%)	15616 (10.1%)	54 (7.06%)
ER			
Negative	10492 (15.9%)	24283 (15.7%)	109 (14.2%)
Positive	55592 (84.1%)	129910 (84.3%)	656 (85.8%)
PR			
Negative	17450 (26.4%)	40867 (26.5%)	216 (28.2%)
Positive	48634 (73.6%)	113326 (73.5%)	549 (71.8%)
HER2			
Negative	56590 (85.6%)	132058 (85.6%)	673 (88.0%)
Positive	9494 (14.4%)	22135 (14.4%)	92 (12.0%)
Side			
Bilateral	9 (0.01%)	25 (0.02%)	0 (0)
Right	32678 (49.4%)	75992 (49.3%)	408 (53.3%)
Left	33397 (50.5%)	78176 (50.7%)	357 (46.7%)
Race			
White	45434 (68.8%)	105605 (68.5%)	0 (0.0%)
Black	13313 (20.1%)	31103 (20.2%)	0 (0.0%)
Others	7337 (11.1%)	17485 (11.3%)	765 (100.0%)
Brain-met			
No	65930 (99.8%)	153836 (99.8%)	745 (97.4%)
Yes	154 (0.23%)	357 (0.23%)	20 (2.61%)

N, number of sample size; ER, estrogen receptor; PR, progesterone receptor; HER2, human epidermal growth factor receptor 2; HR, hormone receptor.

### Univariate and multivariate logistic regression analysis

**FIGURE 1:**
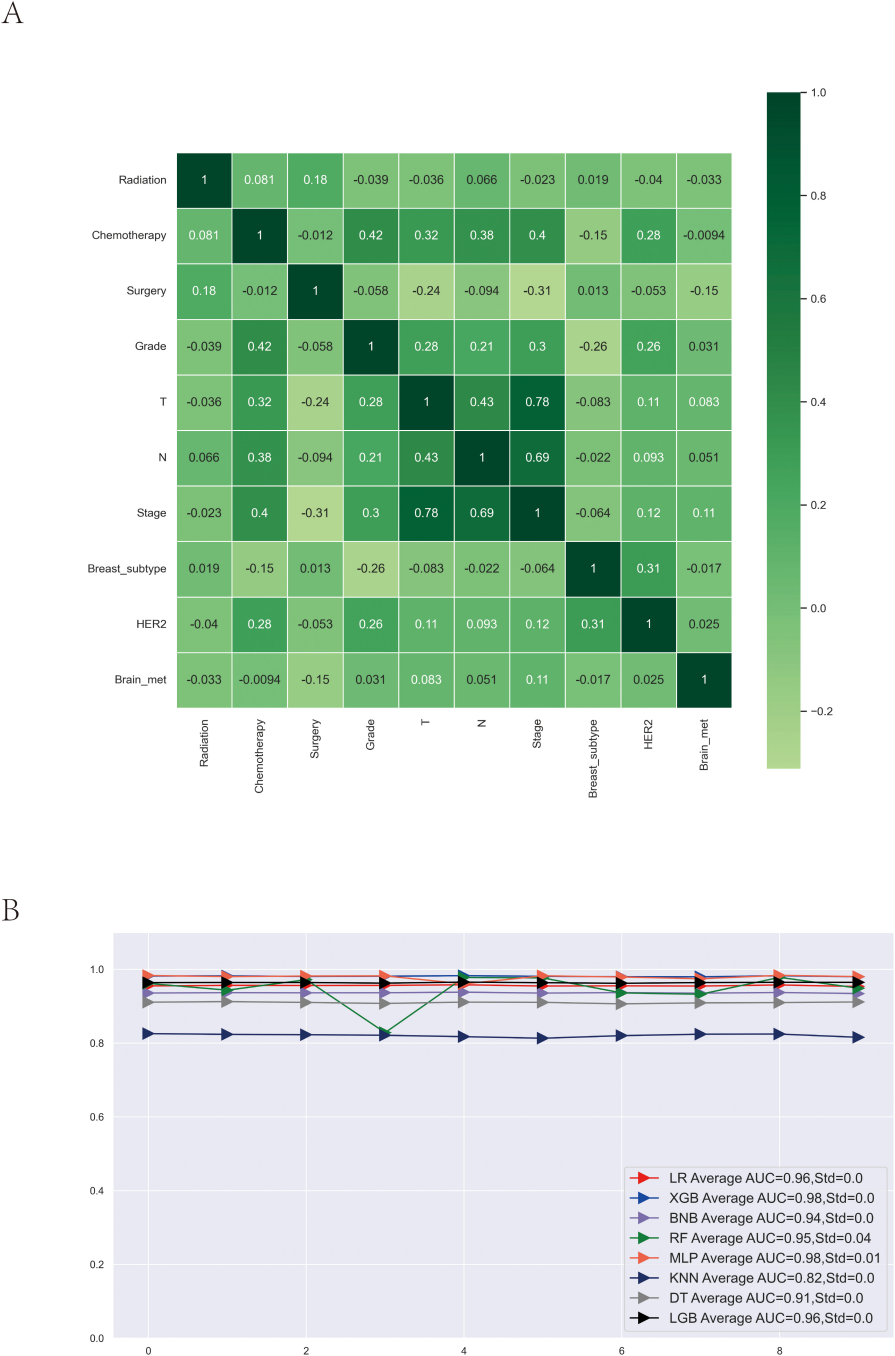
Correlation matrix of clinical variables and comparative performance of eight machine learning algorithms with 10-fold cross-validation for predicting brain metastasis in breast cancer. **(A)** Pearson correlation matrix illustrating the pairwise correlations among clinical and pathological variables. **(B)** AUC values for eight machine learning algorithms obtained through 10-fold cross-validation. ROC, receiver operating characteristic curve; AUC, average area under the curve.

In univariate logistic regression analysis, factors significantly associated with an increased risk of brain metastasis in patients with breast cancer included higher tumor: grade, advanced T stage, lymph node involvement, advanced stage, and PR positivity (Table 2) (*P* < 0.001). Protective factors comprised radiotherapy, chemotherapy, surgery, HR + /HER2- subtype, and unilateral tumors (*P* < 0.001). Age, sex, marital status, ER status, and race showed no significant associations. In multivariate logistic regression analysis, independent risk factors included higher grade, N3 lymph node status, advanced stage, and PR positivity. Protective factors included radiotherapy, chemotherapy, surgery, non-HR-/HER2- subtypes, and HER2 positivity (Table 2). Heatmap analysis revealed no apparent correlations among the included variables (Figure 1A).

**TABLE 2:** Univariate and multivariate logistic regression analysis of risk factors for brain metastasis in breast cancer patients.

Variables	*P*	OR (95%CI)	*P*	OR (95%CI)
Age				
≤50		1.00 (Reference)		
50	0.128	0.85 (0.69∼1.05)		
Sex				
Male		1.00 (Reference)		
Female	0.805	0.88 (0.33∼2.37)		
Radiation				
No		1.00 (Reference)		1.00 (Reference)
Yes	**< 0.001**	0.19 (0.15∼0.25)	**< 0.001**	0.55 (0.43∼0.71)
Chemotherapy				
No		1.00 (Reference)		1.00 (Reference)
Yes	**< 0.001**	0.66 (0.54∼0.79)	**< 0.001**	0.21 (0.17∼0.26)
Marriage				
No		1.00 (Reference)		
Yes	0.595	0.95 (0.80∼1.14)		
Surgery				
No		1.00 (Reference)		1.00 (Reference)
Yes	**< 0.001**	0.02 (0.01∼0.02)	**< 0.001**	0.09 (0.07∼0.12)
Grade				
I		1.00 (Reference)		1.00 (Reference)
II	**< 0.001**	4.76 (3.12∼7.27)	**0.003**	1.94 (1.25∼2.99)
III	**< 0.001**	9.91 (6.53∼15.04)	**< 0.001**	2.41 (1.55∼3.77)
IV	**< 0.001**	75.08 (28.28∼199.31)	**< 0.001**	13.67 (4.69∼39.86)
T				
1		1.00 (Reference)		
2	**< 0.001**	3.85 (2.91∼5.09)		
3	**< 0.001**	11.61 (8.52∼15.82)		
4	**< 0.001**	49.38 (37.86∼64.39)		
N				
0		1.00 (Reference)		1.00 (Reference)
1	**< 0.001**	5.48 (4.43∼6.79)	0.232	1.16 (0.91∼1.47)
2	**< 0.001**	6.63 (4.90∼8.98)	0.570	1.10 (0.78∼1.56)
3	**< 0.001**	14.23 (10.82∼18.73)	**0.015**	1.47 (1.08∼2.01)
Stage				
I		1.00 (Reference)		1.00 (Reference)
II	**< 0.001**	5.91 (3.28∼10.66)	**< 0.001**	4.08 (2.23∼7.47)
III	**< 0.001**	50.96 (29.43∼88.21)	**< 0.001**	33.31 (18.34∼60.52)
IV	**< 0.001**	337.34 (197.31∼576.75)	**< 0.001**	53.63 (29.57∼97.26)
Breast-subtype				
HR-/HER2-		1.00 (Reference)		1.00 (Reference)
HR-/HER2 +	0.187	1.23 (0.90∼1.68)	**< 0.001**	0.06 (0.03∼0.14)
HR + /HER2-	**< 0.001**	0.27 (0.22∼0.33)	**< 0.001**	0.43 (0.31∼0.59)
HR + /HER2 +	0.153	0.82 (0.63∼1.08)	**< 0.001**	0.06 (0.02∼0.14)
ER				
Negative		1.00 (Reference)		
Positive	0.293	1.14 (0.89∼1.47)		
PR				
Negative		1.00 (Reference)		1.00 (Reference)
Positive	**< 0.001**	2.88 (2.39∼3.47)	**< 0.001**	16.98 (7.47∼38.57)
HER2				
Negative		1.00 (Reference)		1.00 (Reference)
Positive	**< 0.001**	0.33 (0.28∼0.39)	**0.037**	0.76 (0.58∼0.98)
Side				
Bilateral		1.00 (Reference)		
Right	**< 0.001**	0.04 (0.01∼0.16)		
Left	**< 0.001**	0.04 (0.01∼0.15)		
Race				
White		1.00 (Reference)		
Black	0.398	1.10 (0.88∼1.36)		
Others	0.625	1.07 (0.81∼1.41)		

N, number of sample size; ER, estrogen receptor; PR, progesterone receptor; HER2, human epidermal growth factor receptor 2; HR, hormone receptor.

### Machine learning model construction

**FIGURE 2:**
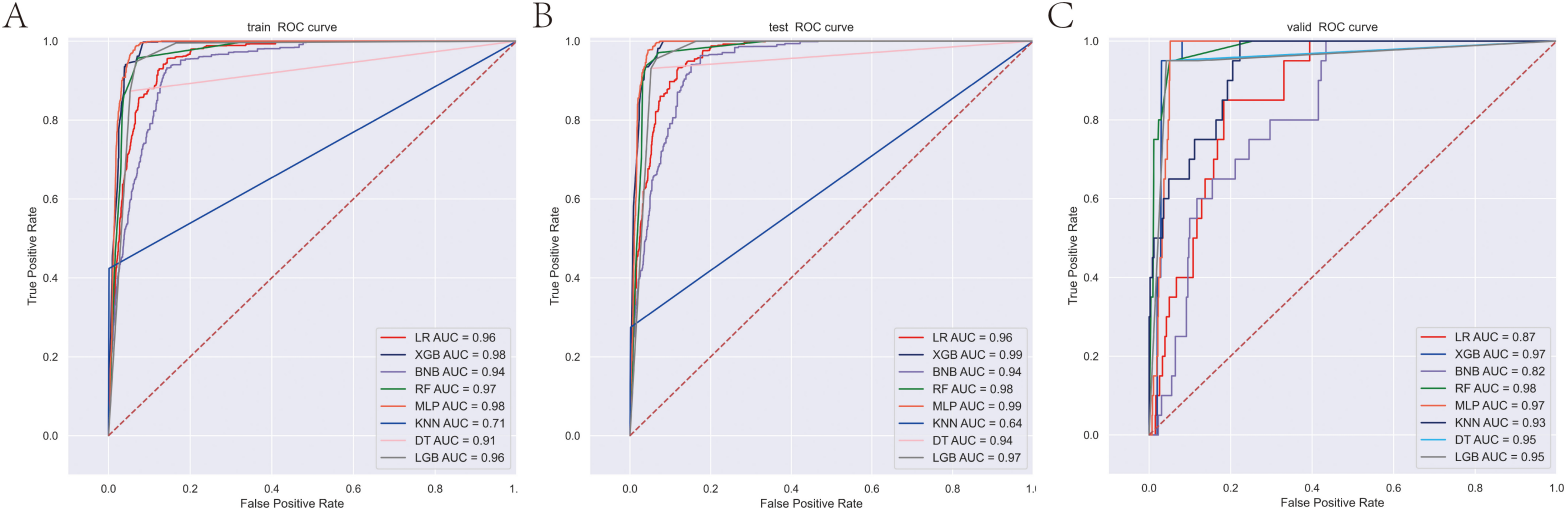
ROC curves of the eight model for predicting brain metastasis in breast cancer across training **(A)**, internal validation **(B)**, and external validation cohorts **(C)**. ROC, receiver operating characteristic curve.

**FIGURE 3:**
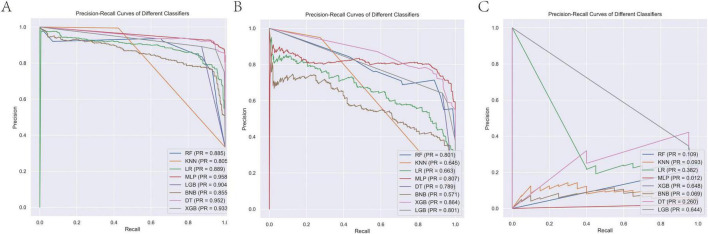
PR curves of the eight model for predicting brain metastasis in breast cancer across training **(A)**, internal validation **(B)**, and external validation cohorts **(C)**. PR, precision-recall curve.

**FIGURE 4:**
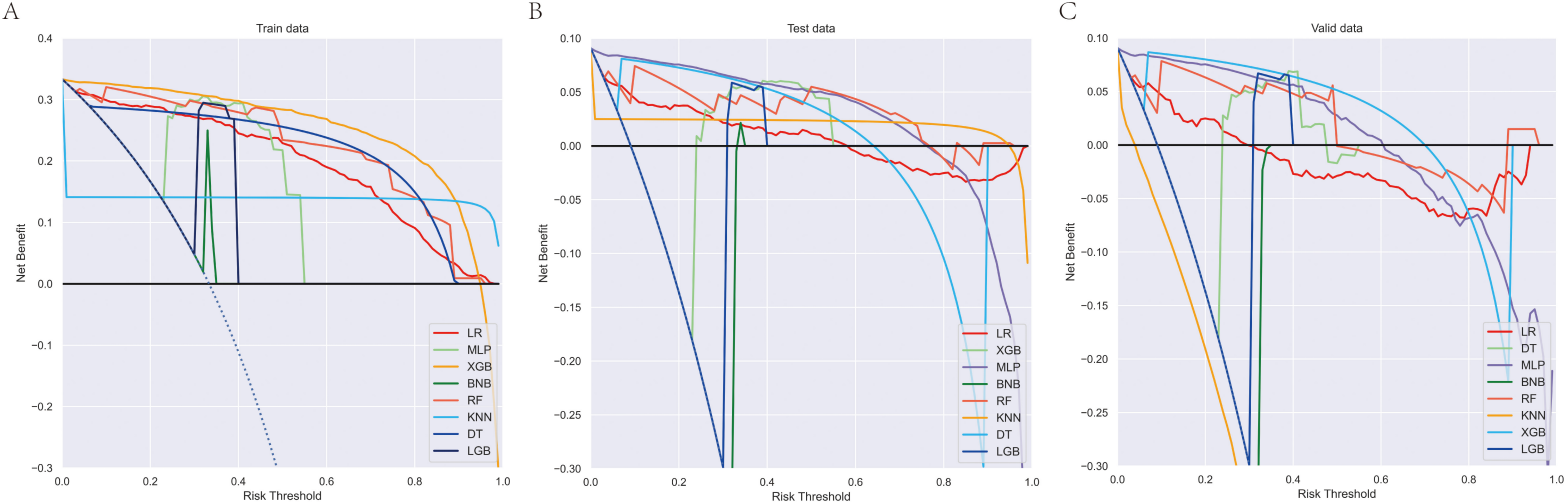
DCA curves of the eight model for predicting brain metastasis in breast cancer across training **(A)**, internal validation **(B)**, and external validation cohorts **(C)**. DCA, decision curve analysis.

**FIGURE 5:**
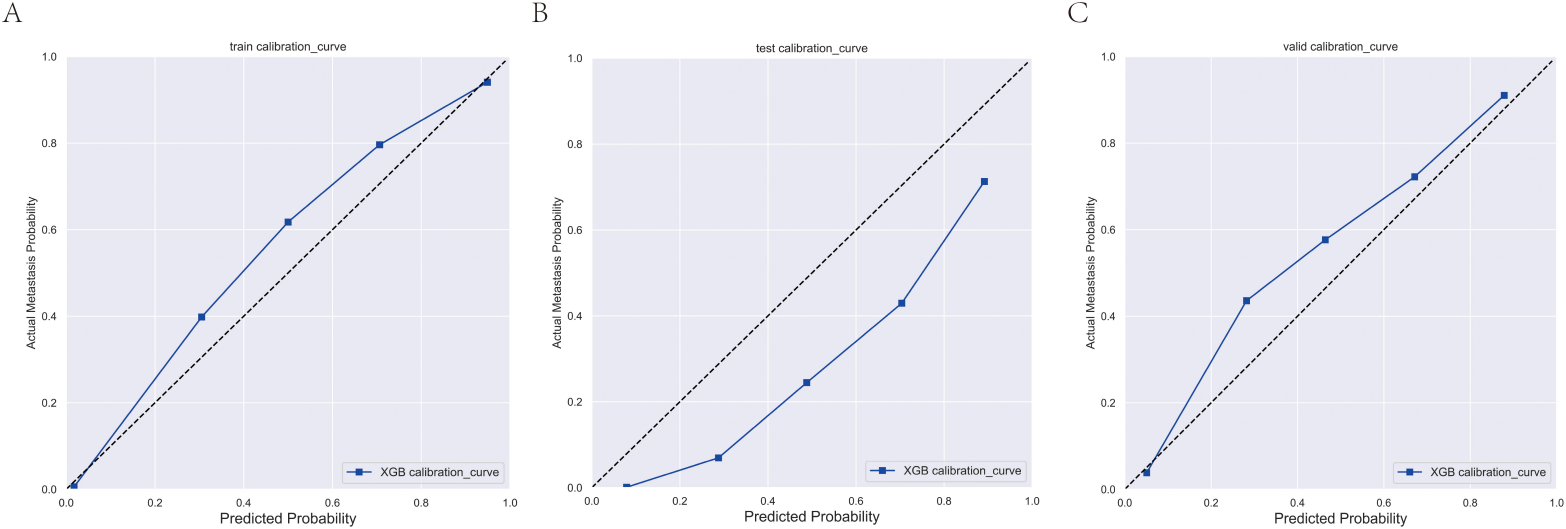
Calibration curves of the XGBoost model for predicting brain metastasis in breast cancer across training **(A)**, internal validation **(B)**, and external validation cohorts **(C)**.

In the present study, the XGB model demonstrated superior and stable predictive performance across diverse datasets. Specifically, in 10-fold cross-validation on the training set, XGB exhibited the highest AUC value (AUC = 0.98, SD = 0.00) (Figure 1B). The AUC values in the training set, internal test set, and external validation set were 0.98, 0.99, and 0.97, respectively, with ROC curves closely hugging the upper-left corner, indicating exceptional discriminatory ability in distinguishing high-risk from low-risk patients (Figures 2A–C). The PR curves further corroborated its advantages in handling imbalanced datasets, with AUPRC values of 0.933, 0.864, and 0.648 in the training set, internal test set, and external validation set, respectively, suggesting a substantial reduction in missed diagnoses during clinical screening for high-risk individuals (Figures 3A–C). Decision curve analysis revealed that, within clinically acceptable risk threshold ranges (0.1–0.8), the net benefit of XGB consistently surpassed that of comparator models across all three cohorts, with particularly pronounced advantages in low-to-moderate threshold intervals; this implies enhanced detection rates for high-risk patients, thereby optimizing clinical decision-making processes (Figures 4A–C). Calibration curve analysis showed excellent agreement between predicted probabilities and observed event rates in the training and external validation sets, with curves nearly overlapping the ideal diagonal line, underscoring the high reliability of the model’s probability outputs. Although a slight underestimation trend was observed in the internal test set within medium-to-high risk intervals, the overall calibration performance remained robust, without evident systematic bias (Figures 5A–C). Collectively, these findings indicate that the XGB model yields highly interpretable and applicable predictions across varying risk levels, supporting its utility as an effective tool for early prediction of brain metastasis risk in breast cancer and providing a reliable foundation for the development of precision intervention strategies.

### Interpretation of machine learning models

**FIGURE 6:**
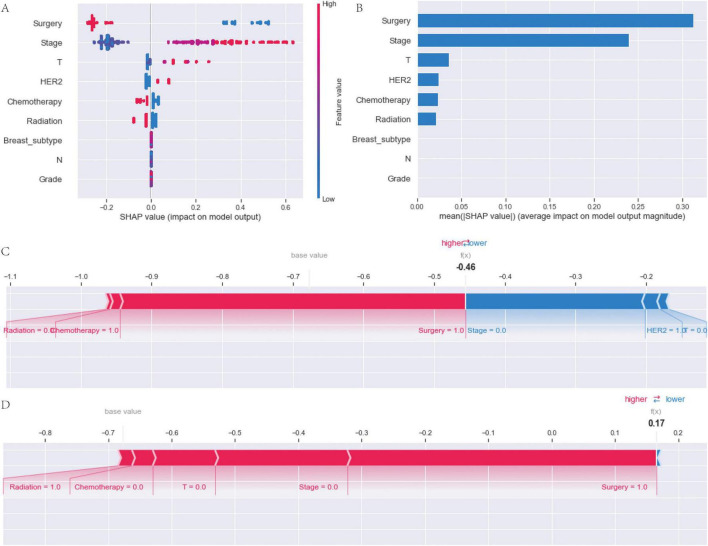
SHAP analysis of feature importance and individual prediction explanations in the breast cancer brain metastasis prediction model. **(A)** SHAP summary plot showing the contribution of each clinical feature to the model output. Each point represents an individual patient. **(B)** Mean absolute SHAP values ranking the overall importance of features in predicting brain metastasis. **(C,D)** SHAP force plots illustrating the contribution of individual features to the predicted risk for two representative patients.

SHAP analysis was employed to quantify the contribution of individual features to the model’s prediction of brain metastasis risk in breast cancer patients (Figures 6A–D). The SHAP bee swarm plot effectively visualized the distribution of SHAP values for each feature, with red and blue color coding indicating an increase or decrease in predicted risk, respectively. Surgical intervention emerged as a significant protective factor; conversely, lower feature values (e.g., the absence of surgical treatment) were associated with higher SHAP values, consequently leading to an increased risk. In contrast, advanced disease Stage and higher T classification consistently demonstrated positive SHAP values, signifying an elevated risk. The mean absolute SHAP value plot, which ranks feature importance based on their average magnitude of impact on the model output, identified surgical intervention as the most influential feature. This was followed by Stage, T stage, HER2 status, chemotherapy, radiation therapy, breast subtype, N stage, and histological grade, in descending order of importance. Furthermore, the SHAP force plot elucidated feature interactions and their cumulative effect on individual predictions. For instance, a scenario characterized by the absence of radiation therapy and the presence of chemotherapy contributed to a reduction in predicted risk [f(x) = -0.46 from the baseline], where surgical intervention and a lower disease stage concurrently exerted protective effects. Conversely, the presence of radiation therapy coupled with the absence of chemotherapy, even in conjunction with low T and N classifications, resulted in an elevated predicted risk [f(x) = 0.17]. This highlights the model’s nuanced sensitivity to treatment-related variables.

### Construction of a web-based calculator

**FIGURE 7:**
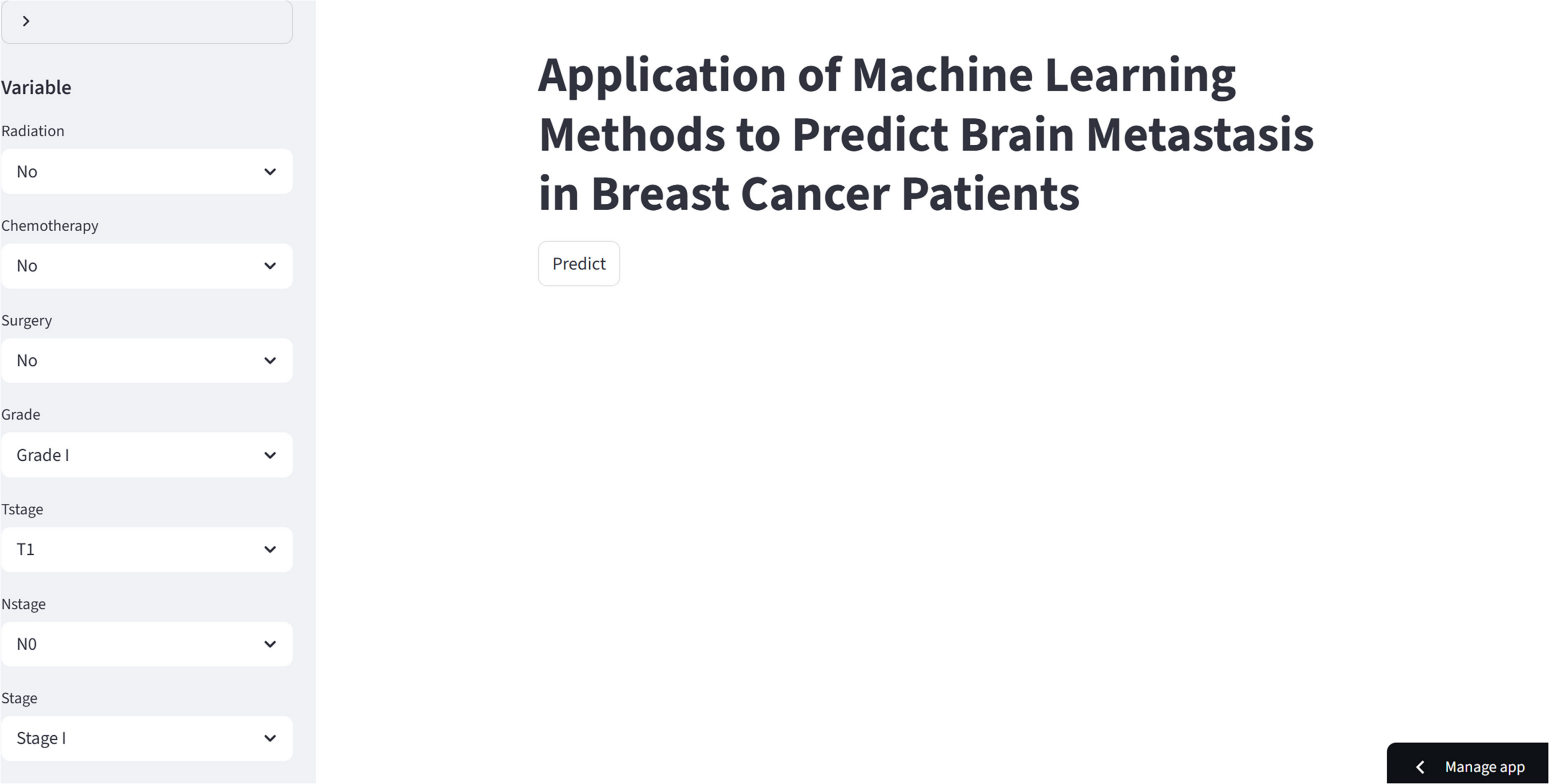
Machine learning application interface for predicting brain metastasis in breast cancer patients (https://p3asu2torgm688ejw6mby5.streamlit.app/).

Based on the XGB algorithm developed above, we have built a convenient web calculator. Users can input the patient’s basic information through the left-side interface to calculate the probability of breast cancer brain metastasis, thereby effectively distinguishing high- and low-risk patients and assisting in clinical promotion and decision-making (Figure 7).

## Discussion

In this study, we developed a predictive model based on the XGB algorithm to forecast the risk of brain metastasis in breast cancer patients. This model incorporated key clinical and pathological factors, including surgery, radiotherapy, chemotherapy, tumor grade, T stage, N stage, clinical stage, HER2 status, and molecular subtype (23). Our model demonstrated excellent performance in identifying high-risk patients, achieved efficient model interpretation through SHAP analysis, and enabled the construction of a user-friendly web-based calculator (24). This facilitates individualized risk stratification while enhancing clinical decision-making.

The treatment-related factors incorporated in the model, such as surgery, radiotherapy, and chemotherapy, highlight their complex roles in predicting brain metastasis. Our analysis revealed a negative correlation between surgical treatment and the occurrence and progression of brain metastasis, which may reflect improved local control of the primary tumor (25, 26). Similarly, chemotherapy—particularly regimens involving anthracyclines and taxanes—has been associated with delayed onset of brain metastasis in certain cohorts, indicating a protective effect against distant progression (27). Furthermore, radiotherapy demonstrated a significant inhibitory effect on breast cancer brain metastasis in this study (28). Some studies suggest that radiotherapy, when used after the diagnosis of brain metastasis, contributes to overall prognosis, often in combination with surgery or stereotactic radiosurgery to extend survival. These findings indicate that aggressive local and systemic therapies can alleviate the burden of extracranial disease, thereby indirectly reducing the incidence of brain metastasis (29, 30). However, the challenge of blood-brain barrier penetration limits the efficacy of chemotherapy against established brain metastases and underscores the necessity of integrating multimodal strategies (31). Moreover, recent research shows that in HER2-positive patients, the combination of chemotherapy and radiotherapy with targeted therapies can significantly improve survival after brain metastasis, further expanding the application potential of these treatment factors in predictive models (32).

Pathological staging factors, including tumor grade, T stage, N stage, and clinical stage, emerged as significant predictors in our XGB model, reflecting their association with aggressive disease progression (33). Poorer tumor grading serves as a strong indicator for brain metastasis, consistent with reports linking lower differentiation to increased metastatic potential (34). Later T stages and N stages similarly predict earlier brain metastasis, as they indicate greater local invasion and lymphatic spread, thereby facilitating distant seeding (35). Overall clinical staging, encompassing metastatic burden and visceral involvement, further amplifies the risk of brain metastasis, with patients having ≥ 2 metastatic sites showing worse outcomes (36). These staging elements enhance the prognostic utility of the model, enabling early identification of patients who may benefit from intensified surveillance or prophylactic interventions. Extending to characteristics of extracranial metastases, such as number, location, and control status, have also been confirmed as important prognostic indicators; for instance, patients with well-controlled extracranial metastases (ECM) exhibit longer survival after brain metastasis, suggesting that future models could further integrate these variables to improve accuracy (37).

HER2 status and molecular subtype are among the most influential features in our model, confirming their critical roles in the pathogenesis of brain metastasis. HER2-positive status strongly predicts an elevated risk of brain metastasis, with incidence rates reaching 35–50% in this subgroup, driven by enhanced tumor aggressiveness and affinity for the central nervous system (38). Molecular subtyping further refines the prediction: triple-negative breast cancer and HER2-enriched subtypes exhibit the highest propensity for brain metastasis and the shortest survival times, whereas hormone receptor-positive subtypes show lower risk and better prognosis. Hormone receptor-negative status exacerbates this risk in HER2-positive cases, accelerating the onset of brain metastasis (39). Targeted therapies, such as trastuzumab, pertuzumab, and tucatinib, have transformed outcomes in HER2-positive brain metastasis, extending survival when incorporated post-diagnosis, which supports the integration of subtype-specific predictions in our model to guide treatment selection (40). Recent systematic reviews further emphasize that the key to prognosis in HER2-positive patients with brain metastasis lies in early screening and multidisciplinary interventions, which aligns closely with our model design (41).

The advantages of the XGB model lie in its ability to handle non-linear interactions among these factors, outperforming traditional logistic regression in terms of accuracy and feature importance analysis. From a clinical perspective, this may facilitate earlier neuroimaging examinations for high-risk patients, thereby improving quality of life by avoiding neurological deficits associated with advanced brain metastasis. Compared to other machine learning models, our XGB model exhibits superior performance on managing imbalanced datasets, as evidenced by higher AUC values and better calibration (42).

Despite these advancements, our study has limitations. The model was trained on retrospective data, which may introduce selection bias and necessitates external validation in diverse cohorts to confirm generalizability. Furthermore, unmeasured confounding factors, such as genetic mutations or lifestyle factors, were not incorporated, potentially limiting the predictive scope. Future research should explore hybrid models integrating imaging data or real-time biomarkers to enhance precision. Additionally, prospective trials evaluating the model’s impact on clinical outcomes, including survival and treatment escalation, are essential. Considering the heterogeneity of brain metastasis, incorporating more ECM-related features, such as metastasis control status.

## Conclusion

In conclusion, our XGB-based predictive model provides a valuable tool for predicting brain metastasis in breast cancer, leveraging established risk factors to guide personalized care. As systemic therapies evolve, integrating such AI-driven predictions may optimize resource allocation and improve patient prognosis in this challenging metastatic setting. Our model not only validates the reliability of these factors but also offers a more comprehensive framework for clinical practice, with anticipation for greater impact in larger-scale prospective validations.

## Data Availability

The original contributions presented in the study are included in the article/Supplementary material, further inquiries can be directed to the corresponding authors.
